# Electrically Reconfigurable Micromirror Array for Direct Spatial Light Modulation of Terahertz Waves over a Bandwidth Wider Than 1 THz

**DOI:** 10.1038/s41598-019-39152-y

**Published:** 2019-02-22

**Authors:** Jan Kappa, Dominik Sokoluk, Steffen Klingel, Corey Shemelya, Egbert Oesterschulze, Marco Rahm

**Affiliations:** 1 Department of Electrical and Computer Engineering, Research Center OPTIMAS, Technische Universität Kaiserslautern, Kaiserslautern, Germany; 2 Department of Experimental Physics, Physics and Technology of Nanostructures, Nano Structuring Center, Technische Universität Kaiserslautern, Kaiserslautern, Germany

## Abstract

We report the design, fabrication and experimental investigation of a spectrally wide-band terahertz spatial light modulator (THz-SLM) based on an array of 768 actuatable mirrors with each having a length of 220 μm and a width of 100 μm. A mirror length of several hundred micrometers is required to reduce diffraction from individual mirrors at terahertz frequencies and to increase the pixel-to-pixel modulation contrast of the THz-SLM. By means of spatially selective actuation, we used the mirror array as reconfigurable grating to spatially modulate terahertz waves in a frequency range from 0.97 THz to 2.28 THz. Over the entire frequency band, the modulation contrast was higher than 50% with a peak modulation contrast of 87% at 1.38 THz. For spatial light modulation, almost arbitrary spatial pixel sizes can be realized by grouping of mirrors that are collectively switched as a pixel. For fabrication of the actuatable mirrors, we exploited the intrinsic residual stress in chrome-copper-chrome multi-layers that forces the mirrors into an upstanding position at an inclination angle of 35°. By applying a bias voltage of 37 V, the mirrors were pulled down to the substrate. By hysteretic switching, we were able to spatially modulate terahertz radiation at arbitrary pixel modulation patterns.

## Introduction

The application of terahertz imaging systems in an industrial environment is currently limited by long recording times for images of larger objects. This is especially true, when additional spectroscopic information is collected during the imaging process.

To overcome these limitations, researchers have been eager during the last two decades to develop new approaches for decreasing the recording times in imaging terahertz spectroscopy^1^. The advancement of this technology is motivated by the properties of the inherently non-ionizing terahertz waves that can be used to screen hazardous material. Especially dielectrics like plastics and textiles are transparent for terahertz radiation. Other substances allow their identification due to strong absorption in the terahertz frequency band. For these reasons, terahertz technology is potentially suited for spectroscopy and imaging of concealed substances in security screening^2,3^,quality control^4–6^ and bio-medical applications^7–9^.

As mentioned above, the use of terahertz imaging systems in an industrial environment is hampered by low power, high cost, and long recording times for imaging the larger objects. The latter plays a crucial role, when additional spectroscopic information is collected during the imaging process^10,11^. Commonly applied imaging techniques include improved raster scanning^11^ and imaging via semiconductor-based focal plane arrays^12,13^, the latter of which do not record spectroscopic data. A more recent approach for increasing data acquisition rates is coded aperture imaging^14^, where a number of various projections of a scene is mapped onto a single-pixel detector. The image is then retrieved by either direct matrix inversion^15^ or by means of compressive computation to further accelerate the reconstruction of the image^16,17^. In all cases, coded aperture imaging requires terahertz spatial light modulators (THz-SLMs) that act as encodable projection aperture grids.

In this context, different concepts for the implementation of THz-SLMs have been reported. While planar metal masks provide THz-SLMs with a high amplitude modulation depth over a wide spectral range, they are not dynamically reconfigurable^16,18^. In contrast, reconfigurable metamaterials accomplish fast dynamic spatial modulation^17,19–24^, yet only for a small spectral working range. This implies that metamaterial SLMs are not necessarily suited for terahertz imaging spectroscopy. Other modulators, as e.g. electrically tunable structures on graphene or vanadium dioxide, ensure a broad spectral working range, but require large pixel sizes^25–28^, which limits the spatial resolution of the images. The concept to dynamically inscribe modulation masks into semiconductors by spatially patterned optical excitation of electrons via commercially available micromirror arrays increases the spatial resolution^29–33^. However, the electromagnetic performance of such modulators is limited by high terahertz intensity loss in the semiconductor itself.

Another interesting approach to modulate amplitude and phase of electromagnetic waves is the application of parity-time-symmetric optical potentials for one-way reflected light^34–36^. This has also been shown for acoustic waves and corresponding parity-time-symmetric acoustic potentials^37,38^.

In the visible and infrared frequency range, micro-electromechanical systems (MEMS) have been established as efficient spatial light modulators. Since such micromirror arrays have proven to be reliable, reconfigurable, spectrally wide-band modulators with high spatial resolution, it would be desirable to use similar types of modulators in the terahertz regime. Though, state-of-the-art micromirror arrays only offer a restricted actuation range that is too small to efficiently interact with terahertz radiation at wavelengths near 100 μm. Moreover, the limited size of the micromirrors causes significant diffraction of terahertz waves and thus decreases the modulation efficiency. At this point, it must be noted that each micromirror in a MEMS-based spatial light modulator constitutes an individual pixel, since the mirror size is larger than the wavelength of light in the optical frequency range. It is obvious that this condition cannot be fulfilled in the terahertz range, where the wavelength at a frequency of 1 THz is 300 µm and the fabrication of mirrors exceeding a size of 300 µm would be extremely challenging. In addition, the electric actuation of such large mirrors would require very high voltages and the movement would be inert. To adress this issue, we recently suggested a new concept for spatial terahertz wave modulation in^39^, where we reported that spectrally wide-band, diffraction-reduced modulation of terahertz radiation can be achieved by reconfigurable gratings that rely on actuable micromirror arrays with high actuation range^40^. Although the minimal mirror lengths in such grating arrays are still of the order of 200 μm to limit diffraction from the mirrors and to guarantee sufficient modulation efficiency, the physical implementation is still extremely challenging. In fact, there are only a few publications dealing with the fabrication of arrays with mirrors of larger size than typically used in the optical range. For example, Canonica *et al*. describe the implementation of large mirror MEMS in reflection geometry^41–43^. However, the reported actuation range of the mirrors is small and thus not suitable for the realization of grating-based large mirror array modulators for terahertz radiation.

In this respect, one promising approach for implementing MEMS with large mirrors is the exploitation of a self assembly method^44–46^. In such a process, a metallic multi-layer structure with differing intrinsic residual stress within and between the individual layers is partially deposited on a sacrificial substrate. Once released from the sacrificial layer, the metallic multi-layer structure bends to compensate for the stress^47^. As a result, one obtains upstanding mirrors that can be moved by application of an electric field due to capacitive effects between mirrors and ground plane. In terahertz technology, the implementation of this concept was succesfully demonstrated in the context of MEMS metamaterials and metasurfaces^48–51^. However, the size of the metallic structures did not exceed 50 μm in reflection systems and therefore was much smaller than the required mirror length of 200 μm. Moreover, the inclination angle of the mirrors was not designed to be usable as reconfigurable reflection grating for spatial terahertz wave modulation.

Here, we report the design, fabrication and experimental evaluation of a grating-based terahertz spatial light modulator that relies on an electro-mechanically reconfigurable array of large mirrors. The reconfigurable grating modulator consists of 4 × 6 independently switchable pixels with a pixel size of 1 mm × 2 mm. Each individual pixel is composed of 4 × 8 micromirrors that either lie flat and form a mirror or are in an inclined position to form a single-pixel grating. The micromirrors are of rectangular geometry with a width of 100 μm and a length of 220 μm. In the upstanding position, the maximum angle between the mirrors of a pixel and the ground electrode plane is 35°. By application of a bias voltage of 37 V, the mirrors of a pixel are completely pulled down and lie flat on the substrate. The broadband spectral working range of the spatial terahertz wave modulator spans from 0.97 THz to 2.28 THz with a modulation depth of more than 50% for normally incident terahertz waves. The achieved peak modulation depth is 87% at 1.38 THz. By means of hysteretic switching, the concept of which is explained in detail in Sec. 3 of the Supplementary Information, it is possible to address each mirror individually such that the terahertz waves can be modulated at arbitrary spatial patterns.

## Fabrication, Design and Modeling

**Figure 1:**
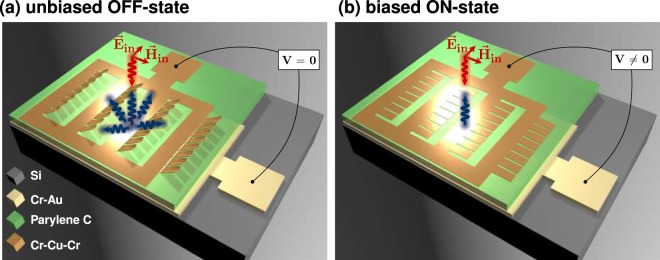
Schematic of a single pixel of the THz-SLM for normally incident terahertz waves. The pixel is composed of mirrors that are arranged in 4 rows and 8 columns. (**a**) OFF-state for a bias voltage of 0 V. All mirrors are inclined and incident terahertz radiation (red) is diffracted away (blue) from the transceiver. (**b**) ON-state for a bias voltage of 37 V. All mirrors are pulled down to the substrate and incident terahertz radiation (red) is reflected (blue) into the transceiver.

It was our goal to implement a grating-based THz-SLM with high modulation depth and wide spectral working range for application in a coded aperture terahertz imaging spectroscope. Under this premise, we asserted by numerical studies that a reconfigurable grating can spatially modulate terahertz waves over a wide spectral range when applied in reflection geometry. The setup of such a modulator and the modulation scheme are illustrated in Fig. 1. The grating modulator consists of an array of mirrors that bend up due to residual strain mismatch in the used material layers (see Fig. 1(a)). In this upstanding case, the mirrors form a diffraction grating for terahertz waves. From the grating of inclined mirrors, normally incident terahertz radiation is diffracted into the different diffraction orders. By design, the grating is devised to suppress back diffraction into the direction of the incoming terahertz beam. This implies that a single-pixel detector or transceiver, placed in this beam path, records a minimum of back-diffracted terahertz waves from the grating. For this reason, we refer to this case as the OFF-state. By application of a bias field, the mirrors can be individually pulled down to lie flat on the substrate (see Fig. 1(b)). In this case, normally incident terahertz radiation is reflected back into the source and a sensor (transceiver) in the beam path detects maximal intensity. For this reason, we refer to this case as the ON-state. Since we can switch the mirrors individually from OFF-state to ON-state and vice versa, we can define discrete pixels of the THz-SLM by grouping *N* mirrors per pixel. In consequence, the minimally achievable pixel size of the modulator is defined by the geometric size of a single mirror. Moreover, we can basically define modulator pixels of arbitrary larger size by grouping multiple mirrors per pixel. By this means, the grating modulator provides a great variety of options to spatially modulate terahertz waves by application of different field bias patterns. In Fig. 1 for example, a discrete modulator pixel is composed of 4 × 8 mirrors that are switched as a group.

The design of the individual mirrors and the pixel-defining groups of mirrors is stipulated by the intended interaction with terahertz radiation. Regarding the design, it must be assured that the mirror size is large enough to avoid immoderate diffraction from individual mirrors, which would blur the spatial modulation contrast between the pixels. We calculated that a minimum mirror length of 200 μm and a minimum width of 100 μm is sufficient to suppress excessive diffraction from individual mirrors in a frequency working range between 0.8 and 2.3 THz, as detailed in the Supplementary Information, Sec. 1.

### Fabrication

**Figure 2:**
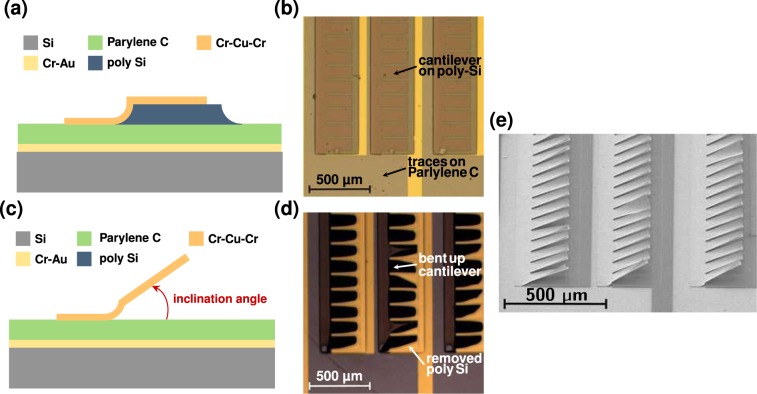
(**a**) Schematic cross-sectional view of an unreleased mirror. The base of the mirror adheres to the parylene C, while the part to be released sits on the poly-Si. (**b**) Microscope image (top view) of the unreleased Cr-Cu-Cr mirrors. The Au ground electrode shines through the parylene C isolation layer. (**c**) Schematic cross-sectional view of a released mirror. The base of the mirror adheres to the parylene C, while the released part is inclined due to residual stress in the Cr-Cu-Cr mirror material. (**d**) Microscope image of the released mirrors. The mirrors are inclined and appear dark under the microscope due to diffraction of light away from the microscope objective. The Au-layer underneath the parylene C is visible. (**e**) SEM image (oblique view) of the inclined mirrors.

For the fabrication of the grating modulator, we first implemented and structured the ground electrode by sputtering a 5 nm thick Cr adhesion layer on a Si-wafer, followed by an Au layer of 100 nm thickness. To electrically insulate the ground electrode from the mirrors, we added a 3 μm parylene C layer as dielectric spacer. Subsequently, we covered the parylene C with 3 μm polycrystalline Si (poly-Si) via plasma-enhanced chemical vapor deposition and structured it during a reactive ion etch process. For implementation of the actuating mirrors, we deposited a Cr(1 nm)-Cu(150 nm)-Cr(8 nm) multi-layer such that the restrained, unmovable base of the mirrors adhered to the parylene C, while the movable mirror parts lay on the structured poly-Si. Furthermore, we evaporated the conducting electrode traces onto the parylene C. In the multi-layer arrangement, the Cu is sealed between the two Cr layers, which prevents oxidization of the Cu. We further structured the Cr-Cu-Cr mirrors by standard photo-lithography to implement the pixelated layout. A schematic cross-sectional view of the layer arrangement is shown in Fig. 2(a) and a microscope picture is depicted in Fig. 2(b). By removal of the poly-Si sacrificial layer via reactive ion etching, we released the parts of the mirrors that incline after losing contact to the poly-Si. After release, the base of the mirrors still adhered to the parylene C, while the strain in the released parts induced significant bending of the mirrors in the vicinity of the base, which brought them in an upstanding position. A schematic cross-sectional view of the layer arrangement and a microscope picture of the released mirrors are shown in Fig. 2(c,d). Additionally, a SEM picture of the released mirrors is depicted in Fig. 2(e).

Each mirror has a length of 220 μm and a width of 100 μm. We determined an average longitudinal inclination angle of 35° with an optical coherence tomograph (see Supplementary Information Sec. 2 or SI-Fig. 6). As mentioned above, the longitudinal inclination is caused by the intrinsic residual stress mismatch between the bottom Cr-Cu layer (tensile) and the top Cr layer (compressive) that must be thicker than the bottom Cr layer^44^. The required individual layer thicknesses for the longitudinal flexure were optimized during multiple fabrication runs. In addition, we observed transverse flexure of the mirrors, which can be seen in Fig. 2(e). The transverse flexure prevents the mirrors from coiling up along the longitudinal direction. For this reason, the longitudinal flexure is mainly confined around the support point at the base of the mirrors and the inclined mirrors are almost straight or only slightly bent along the longitudinal direction. The large inclination angle of the mirrors relative to the substrate enables us to implement grating-based THz-SLMs with large actuation range, as can be seen from Fig. 2(e). Furthermore, the suitable length and width of the mirrors reduces the effect of diffraction from individual mirrors in the terahertz frequency range and thus promises high spatial modulation contrast when used as a THz-SLM.

### Design

For optimization of the THz-SLM, we investigated the dependence of the achievable spatial modulation contrast and the modulation frequency bandwidth on the geometrical size of the mirrors. Hereby, the modulation bandwidth is defined as the spectral bandwidth over which the modulation contrast exceeds a value of 0.5. The modulation contrast describes the ratio *C* = (*R*_ON_ − *R*_OFF_)/(*R*_ON_ + *R*_OFF_), where *R*_ON_ and *R*_OFF_ indicate the corresponding intensity reflectivity for the ON-state and OFF-state, respectively. Furthermore, we determined an optimal number of mirrors that is required to compose a pixel under the pursuit of high spatial resolution and modulation contrast, while keeping the mirror size adequate for fabrication. For the numerical calculations, we used Computer Software Technology (CST) Microwave Studio 2018^©^. Further information on the simulation model and the analysis of the corresponding data can be found in the Supplementary Information Sec. 1. As an optimized design, we implemented a THz-SLM with 24 independently addressable pixels that are composed of 4 × 8 mirrors each. As a result, the grating-based THz-SLM contains 768 actuatable mirrors.

**Figure 3:**
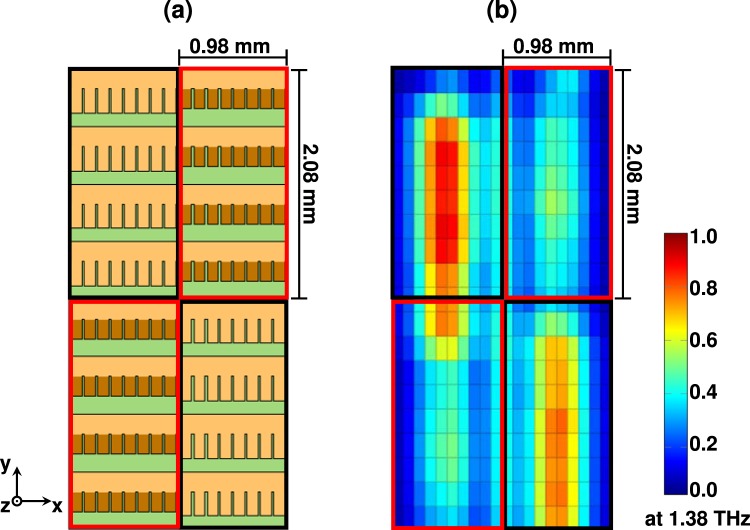
(**a**) Model of a 2 × 2 pixel THz-SLM with two ON-pixels along one diagonal and two OFF-pixels along the other diagonal. ON-pixels are highlighted by black frames, whereas OFF-pixels are marked by red frames. The mirrors in an OFF-pixel are inclined by 35° with respect to the *xy*-plane. (**b**) Spatial distribution of the field intensity of the modulated terahertz waves in a distance of 2 mm from the detector, measured along the *z*-direction. The frequency of the terahertz waves is 1.38 THz. The spatial intensity distribution after diffraction from the THz-SLM agrees well with the coding pattern of the modulator.

In a proof of concept simulation, we numerically calculated the spatial electric field distribution of terahertz waves that were spatially modulated by the THz-SLM. As an example, we calculated the spatial diffraction from a 2 × 2 pixel THz-SLM with two ON-pixels along one diagonal and two OFF-pixels along the other diagonal, as shown in Fig. 3(a). For better visualization, the ON-pixels in Fig. 3(a) are highlighted by black frames, while the OFF-pixels are marked by red frames. The mirrors of the OFF-pixels were homogeneously inclined at 35°. The plane, *x*-polarized terahertz waves were normally incident on the THz-SLM from the −*z*-direction. The electromagnetic boundaries of the calculation domain were open. The magnitude of the diffracted electric field was monitored by field probes that were located in a plane parallel to the *xy*-plane in 2 mm distance from the THz-SLM. The diffracted electric field was recorded in the time domain. We applied a fast fourier transform to calculate the diffracted field intensity at 1.38 THz. The spatial distribution of the diffracted field intensity is depicted in Fig. 3(b). As can be seen, the field intensity of the terahertz waves is spatially modulated according to the modulation pattern illustrated in Fig. 3(a), which evidences the modulation capabilities of the THz-SLM for this example.

### Modeling

**Figure 4:**
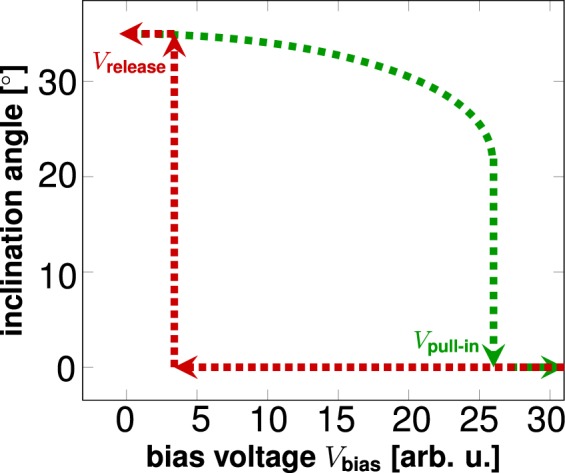
Hysteresis of the mirror actuation as described by a electro-mechanical single beam spring mass model. At *V*_bias_ = 0 V, the mirrors are inclined at the maximal angle. For increasing bias voltage, the inclination angle decreases until the mirrors are pulled in to the substrate at the pull-in voltage *V*_pull-in_. When the bias voltage is decreased again, the mirrors are held down until they snap into the maximally inclined position at the voltage *V*_release_.

In order to theoretically describe the electro-mechanical actuation properties of the THz-SLM, we developed a single beam spring mass model to describe the longitudinal inclination of the mirrors under electrostatic actuation. The derivation of the model is involved and will be published in a separate article. Our mathematical model predicts the dependence of the inclination angle *φ* on the applied bias voltage *V*_bias_ in the manner shown in Fig. 4. The functional dependence reveals a strong voltage-dependent hysteresis of the inclination that allows efficient switching between inclined mirror states and completely flipped-down mirror states. The arrows in Fig. 4 illustrate the hysteretic behavior of the inclination angle for increasing voltages (green dashed arrows) and decreasing voltages (red dotted arrows), respectively. In the hysteretic cycle, the mirrors are inclined for zero voltage. When the voltage is increased, the electrostatic force pulls the mirrors towards the ground plane and the inclination angle continuously decreases to the cusp of the hysteresis curve, where the mirrors are pulled down to the ground electrode at the pull-in voltage *V*_pull-in_. For decreasing voltage, the mirrors are held at the ground plane before being released at the release voltage *V*_release_. At this voltage, the mirrors return to the maximal inclination angle at the start of the hysteresis cycle. In analogy to Canonica *et al*.^41^, we exploited the hysteretic behavior of the mirror inclination for individual, almost independent actuation of all *M* × *N* pixels in the THz-SLM, while reducing the number of required electric contacts to *M* + *N*. In contrast, addressing each pixel by its own contact would require *M* · *N* + 1 contacts. Further explanation of hysteresis-based switching of pixels can be found in the Supplementary Information Sec. 3.

## Experimental Evaluation and Application

In the first test, we investigated the collective behavior of the THz-SLM, which means that all pixels were biased by the same voltage. We determined the hysteresis of the mirror actuation by increasing the voltage, starting from 0 V (mirrors inclined at maximal angle). We observed that all mirrors were pulled down to the ground at a voltage of 37 V. Hereby, the pull-in voltage was not homogeneously distributed over all mirrors, which is owed to differences in the internal stress of individual mirrors. We found the dielectric layer in an insulating state for all our tests, which include bias voltages up to 70 V. Higher voltages were not evaluated. While decreasing the bias voltage starting at 70 V, the mirrors returned to their maximal inclination angle at a release voltage of 15 V, which evidences the expected hysteresis in the mirror actuation.

**Figure 5:**
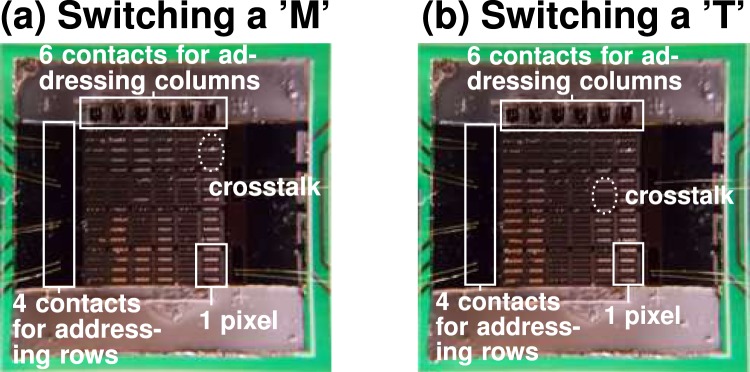
By hysteretic electric switching, individual mirrors and pixels of the THz-SLM can be addressed to display arbitrary patterns. (**a**) The dark pixels display the letter ‘M’. (**b**) The dark pixels display the letter ‘T’.

We utilized the existing hysteresis to create a micro-controller-based switching unit for independent control of the THz-SLM pixels, as described in more detail in Supplementary Information Sec. 3. Figure 5(a,b) show two arbitrary examples of encoded modulation patterns of the THz-SLM. The ‘M’ switched in Fig. 5(a), respectively the ‘T’ in Fig. 5(b), are created by hysteretic electric switching. We observed minor crosstalk between pixels, as highlighted in Fig. 5(a) for the pixel in the upper right corner and in Fig. 5(b) for the fifth pixel from the left in the second row. In addition, we noticed an inhomogeneous distribution of the inclination angles of the individual mirrors within a pixel, which is caused by the self-assembly process. As a direct consequence, the required pull-in voltage for individual mirrors slightly varies within a pixel. Despite this minor inhomogeneity, we were able to determine an average pull-in voltage to switch all mirrors in the pixels with high reliability and absolute reproducibility. Furthermore, we found that deviations of inclination angles between individual mirrors do not deteriorate the modulation efficiency of the THz-SLM. The crosstalk between pixels depends on the relative thicknesses of individual layers in the stacked material of the mirrors^47^. For example, a thicker Cu layer in the Cr-Cu-Cr multi-layer mirrors potentially increases the homogeneity regarding the inclination angle of the mirrors in the THz-SLM, which implies a homogeneous actuation hysteresis for all mirrors and thus a reduction of the cross-talk in hysteretic switching. Furthermore, an increase of the thickness of the top Cr layer for constant adhesion Cr bottom layer thickness increases the inclination angle of the mirrors, which enlarges the difference between the voltages *V*_pull-in_ and *V*_release_ and consequently reduces the crosstalk between pixels as well.

In order to get an estimate on the ON-OFF switching time of the THz-SLM, we applied a periodic rectangular bias voltage with a peak-to-peak voltage of *V*_PP_ = 40 V and a duty-cycle of 50% as a modulation input signal. We visually studied the movement of the mirrors under a microscope and investigated the dependence of the switching behavior on the modulation frequency of the bias voltage. The THz-SLM offered binary switching between ON- and Off-state for frequencies up to ≈1 kHz, which corresponds to a switching time of 1 ms and agrees with the maximal time resolution of the used camera. Unfortunately, higher modulation frequencies could not be measured due to the frequency limit of our measurement equipment.

**Figure 6:**
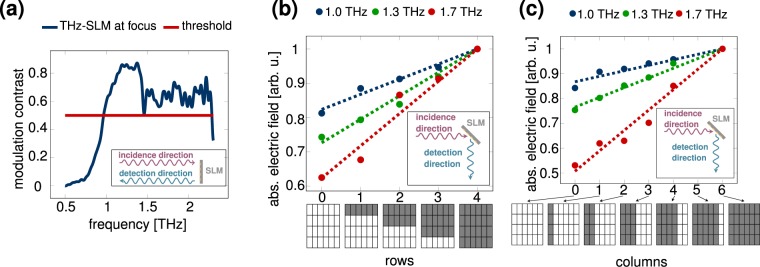
(**a**) Modulation contrast of the THz-SLM. The contrast exceeds a value of 0.5 for a working range from 0.97 THz to 2.28 THz with a maximum contrast of 0.87 at 1.38 THz. (**b**) Linear dependence of the detected modulated electric field on the number of switched-ON rows in the THz-SLM. (**c**) Linear dependence of the detected modulated electric field on the number of switched-ON columns in the THz-SLM.

For experimental evaluation, we measured the modulation contrast and the spectral bandwidth of the THz-SLM using a terahertz time domain spectroscope. The experimental setup is further described in the Supplementary Information Sec. 4. For measuring the contrast between the ON-state and OFF-state of a pixel, we placed the modulator in the focus between two off-axis parabolic mirrors and only illuminated a single pixel. The terahertz radiation was normally incident on the THz-SLM with the electric field polarized perpendicular to the long edge of the mirrors, as shown in Fig. 1. We recorded the reflected terahertz pulse in the time domain and calculated the reflectivity intensity spectrum by application of the fast fourier transform. We then computed the dependence of the modulation contrast on the frequency of the terahertz waves, which is shown in Fig. 6(a). As a reminder, we defined the frequency working range of the THz-SLM as the spectral bandwidth over which the modulation contrast exceeds a threshold of 0.5. Previous studies evidence that such a modulation contrast is sufficient to reconstruct images that are recorded by means of spatial light modulation and single-pixel detection^19,21,29^. With this definition, the modulator offers a spectral working range that spans from 0.97 THz to 2.28 THz with a maximum contrast of 0.87 at 1.38 THz for normally incident terahertz radiation.

In the next step, we examined the applicability of the modulator as reconfigurable grating for spatial terahertz light modulation as described^40^. For this purpose, we illuminated the complete aperture of the grating-based THz-SLM in a collimated terahertz beam and recorded the specular reflection. The THz-SLM was rotated by 45° around the axis along the short edge of the mirrors and was hit by terahertz waves with parallel polarization to this rotation axis. The incidence angle of 45° is close to the Littrow angle, for which we numerically calculated the modulation capabilities of a grating-based modulator in^40^. Unfortunately, the modulator could not be tested at the exact Littrow angle due to restrictions in the optical layout of the measurement setup. To study the dependence between reflected field amplitude into the detector and the number of pixels in the ON-state or OFF-state, we first sequentially increased the number of rows that were switched from the OFF-state to the ON-state and measured the reflected terahertz field amplitude, as depicted in Fig. 5(b). In the schematic 4 × 6 modulator pixel matrix, OFF-pixels are indicated as white, while ON-pixels are indicated as dark. We measured the reflected field amplitude at frequencies of 1.0 THz, 1.3 THz, and 1.7 THz. At all three frequencies, the reflected field amplitude increased proportional to the number of switched-on rows. For reference, the measured reflected field amplitudes were normalized to the case, where all pixels were switched to the ON-state (maximal reflection). In analogy, we observed a linear dependence of the reflected field amplitude on the number of switched-on columns, as shown in Fig. 6(c). Yet, we note that the linear fit agreed better for switching columns than for switching rows. This can be explained by the fundamental property that the spatial width of the diffracted beams in the different diffraction orders becomes more narrow for increasing aperture of the grating along the longitudinal direction of the mirrors. As a consequence, the diffraction orders become spatially more distinguishable. In our setup, the grating aperture is defined by the number of pixel rows. This implies that the aperture length of the grating stays constant at its maximum, when the columns are switched, whereas it varies when the rows are actuated. For this reason, we observe a stricter linearity in the dependence of the detected electric field on the number of switched columns (see Fig. 6(c)) than in the dependence on the number of rows (see Fig. 6(b)).

## Conclusion

We designed, fabricated and experimentally demonstrated modulation of terahertz waves over a frequency range from 0.97 THz to 2.28 THz. Over the entire frequency band, the modulation contrast exceeded a value of 0.5 with a maximal contrast of 0.87 at 1.38 THz. The terahertz spatial light modulator (THz-SLM) consisted of 768 actuatable mirrors with a length of 220 μm and a width of 100 μm. Such large mirror sizes are required to reduce diffraction from individual mirrors, which would limit the pixel-to-pixel modulation contrast. The minimum pixel size of the modulator is dictated by the mirror size, while larger pixel sizes can be readily defined by grouping of mirrors that are steered simultaneously. We applied the mirror array as a reconfigurable grating for spatial modulation of terahertz waves that are diffracted from the reflection grating. For fabrication, we exploited residual stress in the mirrors that consist of chrome-copper-chrome multi-layers with a total thickness of 159 nm. The internal tension forces the mirrors to an inclined position at an angle of 35°. By applying a bias voltage of 37 V between mirror and ground plane, the mirrors were completely pulled down to the substrate. By analytic modeling, we determined the hysteresis of the mechanical movement of the mirrors and exploited it for addressing individual pixels by hysteretic electric switching via a low-cost micro-controller. By this means, we were able to modulate terahertz waves at arbitrary pixel patterns. Due to its large operating bandwidth, the modulator is perfectly suited as THz-SLM in a coded aperture imaging spectroscope.

## Supplementary information

Supplementary Information to Electrically Reconfigurable Micromirror Array for Direct Spatial Light Modulation of Terahertz Waves over a Bandwidth Wider Than 1 THz

## References

[1] Mittleman DM (2018). Twenty years of terahertz imaging [invited]. Opt. Express.

[2] Federici JF (2005). Thz imaging and sensing for security applications—explosives, weapons and drugs. Semiconductor Science and Technology.

[3] Cooper KB (2011). Thz imaging radar for standoff personnel screening. IEEE Transactions on Terahertz Science and Technology.

[4] Rutz F (2006). Terahertz quality control of polymeric products. International Journal of Infrared and Millimeter Waves.

[5] Ahi, K., Shahbazmohamadi, S. & Asadizanjani, N. Quality control and authentication of packaged integrated circuits using enhanced-spatial-resolution terahertz time-domain spectroscopy and imaging. *Optics and Lasers in Engineering* **104**, 274–284, 10.1016/j.optlaseng.2017.07.007, Optical Tools for Metrology, Imaging and Diagnostics (2018).

[6] Wang C, Zhou R, Huang Y, Xie L, Ying Y (2019). Terahertz spectroscopic imaging with discriminant analysis for detecting foreign materials among sausages. Food Control.

[7] Nazarov MM, Shkurinov AP, Kuleshov EA, Tuchin VV (2008). Terahertz time-domain spectroscopy of biological tissues. Quantum Electronics.

[8] Gallerano GP, Park G-S, Ramundo-Orlando A, Zeni O (2018). Guest editorial: Special issue on thz radiation applied to biophysical, biological, and biomedical sciences. Journal of Infrared, Millimeter, and Terahertz Waves.

[9] Chavez T, Bowman T, Wu J, Bailey K, El-Shenawee M (2018). Assessment of terahertz imaging for excised breast cancer tumors with image morphing. Journal of Infrared, Millimeter, and Terahertz Waves.

[10] Schumann S (2012). Spectrum to space transformed fast terahertz imaging. Opt. Express.

[11] Kanda N, Konishi K, Nemoto N, Midorikawa K, Kuwata-Gonokami M (2016). Real-time broadband terahertz spectroscopic imaging by using a high-sensitivity terahertz camera. Scientific Reports.

[12] Trichopoulos GC, Mosbacker HL, Burdette D, Sertel K (2013). A broadband focal plane array camera for real-time thz imaging applications. IEEE Transactions on Antennas and Propagation.

[13] Schuster F (2011). Broadband terahertz imaging with highly sensitive silicon cmos detectors. Opt. Express.

[14] Kannegulla A (2014). Coded-aperture imaging using photo-induced reconfigurable aperture arrays for mapping terahertz beams. IEEE Transactions on Terahertz Science and Technology.

[15] Augustin S, Frohmann S, Jung P, Hübers H-W (2018). Mask responses for single-pixel terahertz imaging. Scientific Reports.

[16] Chan WL (2008). A single-pixel terahertz imaging system based on compressed sensing. Applied Physics Letters.

[17] Watts CM (2014). Terahertz compressive imaging with metamaterial spatial light modulators. Nat Photon.

[18] Ma Y, Grant J, Saha S, Cumming DRS (2012). Terahertz single pixel imaging based on a nipkow disk. Opt. Lett..

[19] Chan WL (2009). A spatial light modulator for terahertz beams. Applied Physics Letters.

[20] Shrekenhamer D, Chen W-C, Padilla WJ (2013). Liquid crystal tunable metamaterial absorber. Physical review letters.

[21] David, S., John, M., Sanjay, K. & J., P. W. Four color metamaterial absorber thz spatial light modulator. *Advanced Optical Materials* **1**, 905–909, 10.1002/adom.201300265.

[22] Liu M (2017). Ultrathin tunable terahertz absorber based on mems-driven metamaterial. Microsystems &Amp; Nanoengineering.

[23] Rout S, Sonkusale SR (2016). A low-voltage high-speed terahertz spatial light modulator using active metamaterial. APL Photonics.

[24] Hu F (2016). A dynamically tunable terahertz metamaterial absorber based on an electrostatic mems actuator and electrical dipole resonator array. Journal of Micromechanics and Microengineering.

[25] Kakenov N (2015). Graphene-enabled electrically controlled terahertz spatial light modulators. Opt. Lett..

[26] Fu M (2017). Efficient terahertz modulator based on photoexcited graphene. Optical Materials.

[27] Mittendorff M, Li S, Murphy TE (2017). Graphene-based waveguide-integrated terahertz modulator. ACS Photonics.

[28] Hoque MNF, Karaoglan-Bebek G, Holtz M, Bernussi AA, Fan Z (2015). High performance spatial light modulators for terahertz applications. Optics Communications.

[29] Shrekenhamer D, Watts CM, Padilla WJ (2013). Terahertz single pixel imaging with an optically controlled dynamic spatial light modulator. Opt. Express.

[30] Mohr T, Herdt A, Elsässer W (2018). 2d tomographic terahertz imaging using a single pixel detector. Opt. Express.

[31] Stantchev, R. I. *et al*. Noninvasive, near-field terahertz imaging of hidden objects using a single-pixel detector. *Science Advances* **2**, 10.1126/sciadv.1600190, http://advances.sciencemag.org/content/2/6/e1600190.full.pdf (2016). 10.1126/sciadv.1600190 PMC4928995 27386577

[32] Xie Z (2013). Spatial terahertz modulator. Scientific Reports.

[33] He JW (2017). Reconfigurable terahertz grating with enhanced transmission of te polarized light. APL Photonics.

[34] Zhu X-F (2015). Defect states and exceptional point splitting in the band gaps of one-dimensional parity-time lattices. Opt. Express.

[35] Zhu X-F, Peng Y-G, Zhao D-G (2014). Anisotropic reflection oscillation in periodic multilayer structures of parity-time symmetry. Opt. Express.

[36] Zhu X, Feng L, Zhang P, Yin X, Zhang X (2013). One-way invisible cloak using parity-time symmetric transformation optics. Opt. Lett..

[37] Liu T, Zhu X, Chen F, Liang S, Zhu J (2018). Unidirectional wave vector manipulation in two-dimensional space with an all passive acoustic parity-time-symmetric metamaterials crystal. Phys. Rev. Lett..

[38] Zhu X, Ramezani H, Shi C, Zhu J, Zhang X (2014). *PT*-symmetric acoustics. Phys. Rev. X.

[39] Schmitt KM, Rahm M (2016). Evaluation of the impact of diffraction on image reconstruction in single-pixel imaging systems. Opt. Express.

[40] Kappa J, Schmitt KM, Rahm M (2017). Electromagnetic behavior of spatial terahertz wave modulators based on reconfigurable micromirror gratings in littrow configuration. Opt. Express.

[41] Canonica MD, Zamkotsian F, Lanzoni P, Noell W, de Rooij N (2013). The two-dimensional array of 2048 tilting micromirrors for astronomical spectroscopy. Journal of Micromechanics and Microengineering.

[42] Canonica, M. *et al*. Large micromirror array for multi-object spectroscopy in a cryogenic environment (2009).

[43] Canonica MD, Zamkotsian F, Lanzoni P, Noell W, Rooij ND (2013). Optical characterization of two-dimensional array of 2048 tilting micromirrors for astronomical spectroscopy. Opt. Express.

[44] Tyagi P (2009). Self-assembly based on chromium/copper bilayers. Journal of Microelectromechanical Systems.

[45] Monnai, Y. *et al*. Terahertz beam steering using structured mems surfaces for networked wireless sensing. In *2012 Ninth International Conference on Networked Sensing* (*INSS*), 1–3, 10.1109/INSS.2012.6240544 (2012).

[46] Hillmer H (2018). Optical mems-based micromirror arrays for active light steering in smart windows. Japanese Journal of Applied Physics.

[47] Chen C (2017). Quantitative analysis and predictive engineering of self-rolling of nanomembranes under anisotropic mismatch strain. Nanotechnology.

[48] Pitchappa P, Ho CP, Dhakar L, Lee C (2015). Microelectromechanically reconfigurable interpixelated metamaterial for independent tuning of multiple resonances at terahertz spectral region. Optica.

[49] Pitchappa, P. *et al*. Microelectromechanically tunable multiband metamaterial with preserved isotropy. *Scientific Reports* (2015). 10.1038/srep11678 PMC4481855 26115416

[50] Shih K (2017). Active mems metamaterials for thz bandwidth control. Applied Physics Letters.

[51] Longqing, C. *et al*. Active multifunctional microelectromechanical system metadevices: Applications in polarization control, wavefront deflection, and holograms. *Advanced Optical Materials* **5**, 1600716, 10.1002/adom.201600716.

