# Supplementary material for: Electrically Reconfigurable Micromirror Array for Direct Spatial Light Modulation of Terahertz Waves over a Bandwidth Wider Than 1 THz
Source: Sci Rep. 2019 Feb 22;9:2597. doi: 10.1038/s41598-019-39152-y (PMC6385255; doi:10.1038/s41598-019-39152-y)
Supplement: Supplementary file 1 — Supplementary Information to Electrically Reconfigurable Micromirror Array for Direct Spatial Light Modulation of Terahertz Waves over a Bandwidth Wider Than 1 THz [file 41598_2019_39152_MOESM1_ESM.pdf]

Supplementary Information to

Electrically Reconfigurable Micromirror Array for Direct Spatial Light Modulation of Terahertz Waves over a Bandwidth Wider Than 1 THz

Jan Kappa[1,*], Dominik Sokoluk[1], Steffen Klingel[2], Corey Shemelya[1], Egbert Oesterschulze[2], and Marco Rahm[1]

[1]Department of Electrical and Computer Engineering, Research Center OPTIMAS, Technische Universität Kaiserslautern, Kaiserslautern, Germany
[2]Department of Experimental Physics, Physics and Technology of Nanostructures, Nano Structuring Center, Technische Universität Kaiserslautern, Kaiserslautern, Germany
[*]kappa@eit.uni-kl.de

This PDF file includes:

- Supplementary text on the following subjects:
  1. Simulation Model
  2. Stress-Induced Bending of the Mirrors
  3. Hysteretic Actuation Concept
  4. Experiment
- Supplementary Figures:
  - SI-Fig. 1 to SI-Fig. 7
- Supplementary Equations:
  - SI-1
  - SI-2

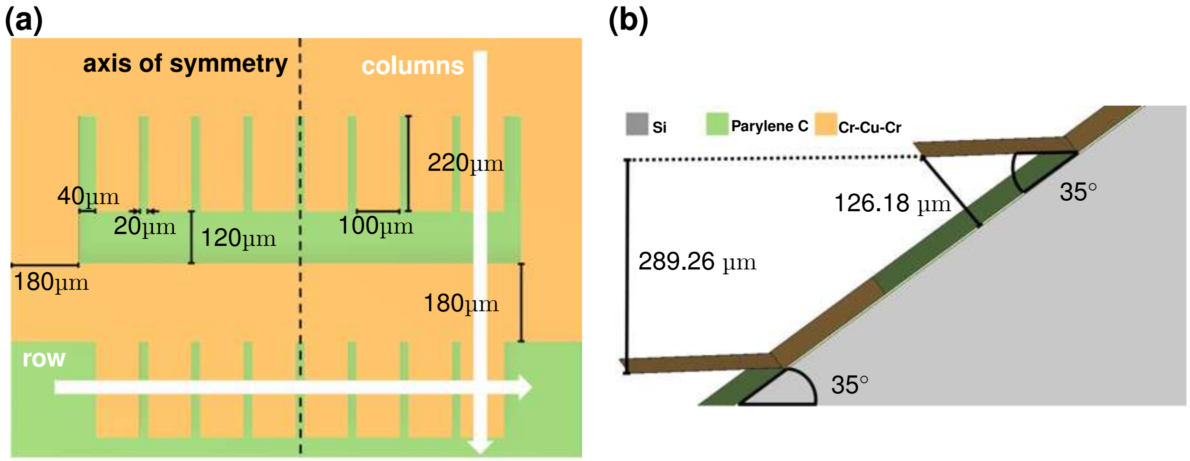

SI-Fig. 1. (a) Schematic top view of the mirrors and corresponding physical dimensions as used in the experiment. The mirrors are arranged in a rectangular matrix consisting of rows and columns. (b) Schematic side view of the modulator in Littrow configuration and corresponding inclination angles, distance between mirror tip and substrate and vertical distance between mirrors.

1 Simulation Model

As explained in the main article, the implemented terahertz spatial light modulator (THz-SLM) follows the principle of a reconfigurable grating. The grating is composed of mirrors, whose inclination can be independently switched between two states, thus providing us with the capability of realizing arbitrary digitized grating patterns for light modulation. We distinguish between the ON-state and the OFF-state of the individual mirrors. A mirror is in the ON-state, when it reflects incoming radiation to the detector and is in the Off-state, when it diffracts the radiation away from the detector.
Such a grating-based THz-SLM can be implemented in two different configurations. In the Littrow configuration, the base-plane of the grating is rotated exactly by the maximal inclination angle of the mirrors (see SI-Fig. 2). Terahertz radiation is then normally incident onto the mirrors, when they are in the OFF-state (inclined at the maximal inclination angle) and the radiation is reflected back into the source. When the mirrors are in the ON-state (flat on the substrate), terahertz radiation is incident under the rotation angle of the grating and is reflected into the detector in accordance with the reflection law. In the normal incidence configuration, the base plane of the grating is normal to the incident terahertz radiation (see SI-Fig. 3). In this case, source and receiver are at the same position (transceiver). The mirrors of the grating are in the ON-state, when they lie flat on the substrate and incident radiation is reflected back into the transceiver. The mirrors are in the OFF-state, when they are inclined at the maximal angle and incident radiation is diffracted away from the detector.
For spatial light modulation, the minimum pixel size is defined by the size of a single mirror. Larger pixel sizes can be realized by grouping multiple mirrors into one pixel. For maximization of the spatial modulation contrast, we determined the optimal pixel size of the grating-based THz-SLM by means of numerical calculations. For later comparison with the electromagnetic properties of the modulator in the experiment, we accounted for constraints of the applied fabrication process in the simulation model. In good approximation, we neglected the transverse flexure of the mirrors, which rarely impacts the modulation contrast.

The physical size of the mirrors in the simulation was identical to the implemented size in the fabricated mirror array. The geometry and the dimensions of the mirrors are shown in SI-Fig. 1a). As measured in the experiment, the mirrors in the numerical model inclined to a maximal angle of 35°, as shown in SI-Fig. 1b).
For the Littrow configuration in SI-Fig. 2, we defined the terahertz source by a waveguide port. The base line of the grating was rotated counterclockwise by 35°. We applied perfect electric boundary conditions in the x-y-plane, which defines the polarization of the incident radiation in z-direction. For the other planes, we applied open boundary conditions. Although the boundary conditions define a mirror array that is extended to infinity in the z-direction, the investigated configuration is well suited for a first test of the spectral dependence of the modulation contrast of the THz-SLM on the number of mirror rows in a pixel. At the same time, the required calculation time is held at a minimum. The detector was implemented by an array of field probes. To determine the spectral reflectivity of the grating, we spatially averaged the square amplitude of the fourier transform of the electric field over the field probes. Hereby, the phase of the electric field was nearly constant along the field probe positions.

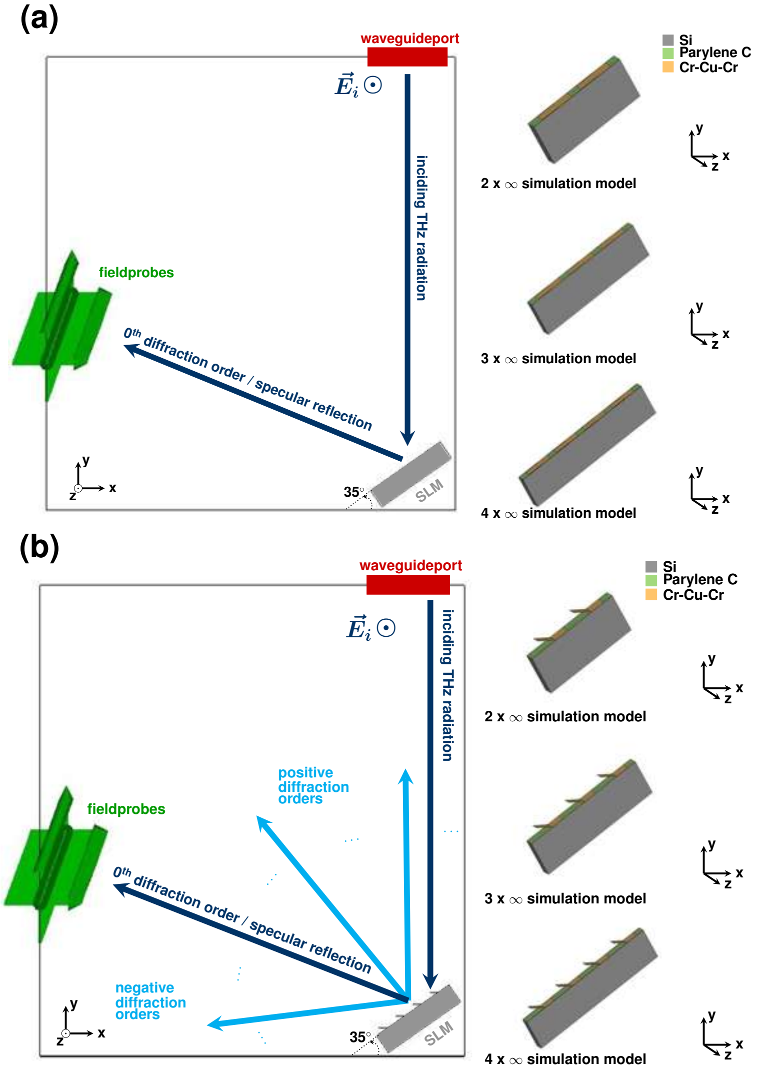

SI-Fig. 2. Simulation model for reconfigurable grating in Littrow-configuration (perfect electric conductor boundary in z-direction, open boundaries otherwise). (a) ON-state state, for which incident radiation is reflected into the detector, (b) OFF-state, for which incident radiation is diffracted away from the detector.

For the normal incidence configuration in SI-Fig. 3, the boundary conditions and the field polarization were kept similar to the definitions in the Littrow configuration. The transceiver was modeled by a waveguide port, which rendered the use of field monitors obsolete.

SI-Fig. 4a) shows the dependence of the modulation contrast on the terahertz frequency for increasing number of rows in a pixel for the normal incidence configuration.

Hereby, the modulation contrast is defined as $C = (R_{\mathrm{ON}} - R_{\mathrm{OFF}})/(R_{\mathrm{ON}} + R_{\mathrm{OFF}})$, where R_{ON} denotes the reflectivity of the THZ-SLM for all pixels switch to the ON-state and R_{OFF} is the reflectivity for all pixels in the OFF-state. The reflectivity is defined with respect to the terahertz wave intensity. In the following, we denote the arrangement of mirrors in a pixel by a $N \times M$ sub-matrix, where N denotes the number of mirror rows and M the number of mirror columns. As reported in the literature[1], a minimum modulation contrast of about 0.4 is sufficient to reconstruct images that are recorded via coded aperture imaging. For this reason, we define the working range of the THz-SLM as the spectral bandwidth, within which the modulation contrast exceeds a value 0.5. As can be seen in SI-Fig. 4(a), the modulation contrast slightly increases with the number of rows in a pixel. While the contrast for the $2 \times \infty$-pixels achieves a maximal value of 0.61 at a frequency of 0.75 THz, the $3 \times \infty$- and $4 \times \infty$-pixels allow maximum contrasts of 0.72 at 1.6 THz and 0.71 at 1.6 THz, respectively . The broadest spectral working range was observed for the $4 \times \infty$-pixel modulator with a range between 0.8 THz and 2.3 THz. With the exception of narrow

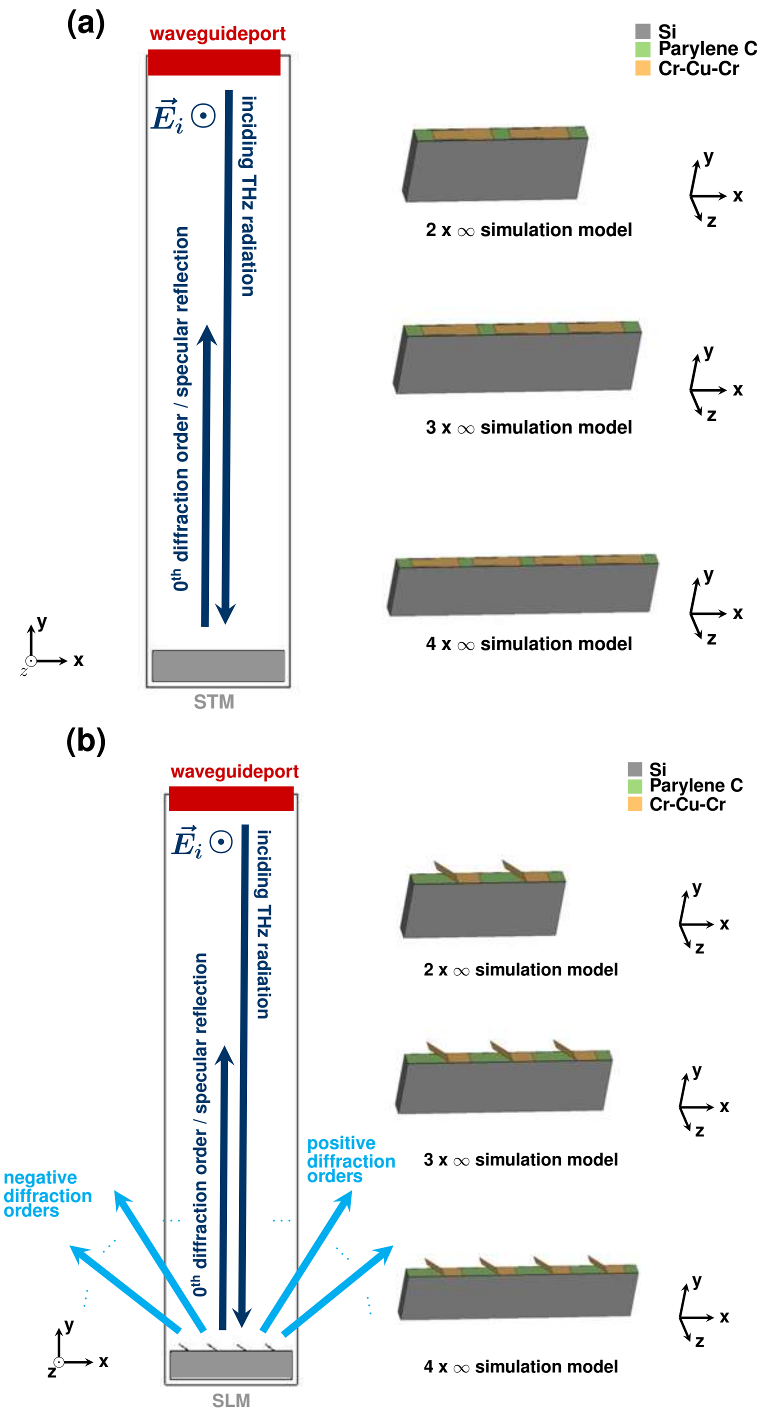

SI-Fig. 3. Simulation model for reconfigurable grating in normal incidence configuration (perfect electric conductor boundary in z-direction, open boundaries otherwise). (a) ON-state state, for which incident radiation is reflected into the detector (transceiver), (b) OFF-state, for which incident radiation is diffracted away from the detector (transceiver).

frequency bands between 0.94 THz and 1.18 THz as well as between 1.65 THz and 1.83 THz, the modulation contrast exceeds a value of 0.5 and is permanently higher than 0.4.

As explained in[2], the highest modulation contrast of grating-based THz-SLMs can be achieved in the Littrow configuration. In such a setup, the mirrors lie flat on the substrate in the ON-state and the grating acts as a flat mirror with maximum reflection

into the detector. In the OFF-state, the mirrors are normal to the incident radiation and the grating mainly diffracts into the source (away from the detector). The field amplitudes of the diffracted waves into the source direction display distinct maxima for frequencies f_m that obey the equation

$$f_m = m\frac{c_0}{2\Delta s} \quad \text{(SI-1)}$$

Hereby, m indicates the diffraction order and c_0 the velocity of light in vacuum. The spectral distribution of the directional diffraction efficiency of the grating into the direction of source is depicted in SI-Fig. 4(c). The directional diffraction efficiency describes the ratio between diffracted intensity of a wave into a defined direction and the intensity of the incident wave on the grating. As predicted by SI-1, the diffraction maxima are observed at frequencies f_m. The diffraction efficiency increases for increasing number of mirror rows in the pixel. In comparison with the normal incidence configuration, the spectral working range of the THz-SLM blue-shifts to a range between 1.75 THz and 3.0 THz. This can be readily explained by the larger groove depth of the THz-SLM, which amounts to 298.6 µm for the Littrow configuration in comparison with 126.18 µm for the normal incidence configuration. As for the normal incidence configuration, the modulation contrast increases for increasing number of mirror rows in the pixel. We determined a maximum contrast of 0.9 at 2.38 THz for $4\times\infty$ mirror pixels. The relative spectral working range of the THz-SLM (spectral range divided by center frequency) in Littrow configuration was 1.9 and thus slightly larger than the relative working range of the THz-SLM of 1.03 in the normal incidence configuration.

After studying the spectral properties of the modulation contrast for pixels that are comprised of a finite number of rows in the x-direction and an infinite number of columns in the z-direction, we investigated the impact of limiting the number of mirror columns in a pixel on the diffraction properties and the modulation contrast. As explained above, the numerical calculation of electric fields diffracted from 2-D confined pixels requires exorbitant calculation time. For this reason, we only investigated the far-field diffraction of such THz-SLMs and the corresponding modulation contrast at four exemplary frequencies, namely 1.6 THz and 2.0 THz in the normal incidence case and 2.0 THz and 2.4 THz for the Littrow configuration . After a series of simulations, we determined that pixels comprised of 4×8 mirrors are of suitable size for achieving a high modulation contrast. SI-Fig. 5(a) and 5(b) illustrate the model for the numerical calculation of the reflected (diffracted) electric far fields and the modulation contrast of the THz-SLM for the normal incidence and the Littrow configuration, respectively. In both scenarios, we applied open conditions for all boundaries and investigated the diffraction of plane waves with parallel-polarization from the grating (electric field vector parallel to the short edges of the mirrors). The plane waves are incident from $z=-\infty$. In the Littrow configuration in SI-Fig. 5b), the base plane of the grating is rotated by 35° around the axis parallel to the short edge of the mirrors.

From the 3-D full wave simulations, we extracted the electric far-field distribution of the reflected (diffracted) fields in the y-z cutting plane ($\phi = 90°$ in SI-Fig. 5(a) and 5(b)) and plotted the dependence on the diffraction angle θ that is measured against the z-axis and spans from -90° to +90° to cover the upper half space in the y-z-plane of SI-Fig. 5(a) and 5(b). SI-Fig. 5(c) and 5(e) depict the electric far field for the normal incidence configuration at frequencies of 1.6 THz and 2.0 THz, respectively.

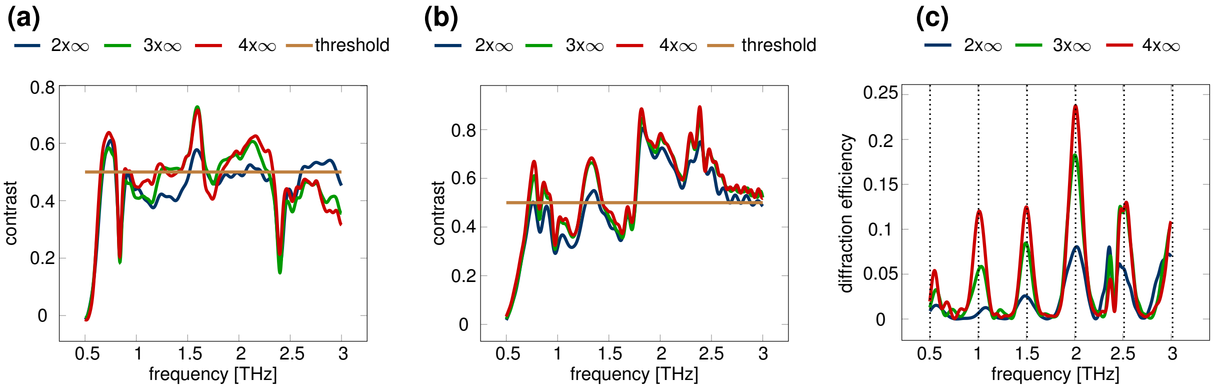

SI-Fig. 4. Simulated modulation contrast for switching between all pixels in the ON-state and all pixels in the OFF-state. In the plots, the modulation contrast was evaluated for varying number of mirror rows in a pixel, while the number of columns was infinite. (a) Calculated modulation contrast for normal incidence configuration. (b) Calculated modulation contrast for Littrow configuration. (c) Calculated diffraction efficiency in Littrow configuration for varying number of mirror rows and infinite number of columns in a pixel.

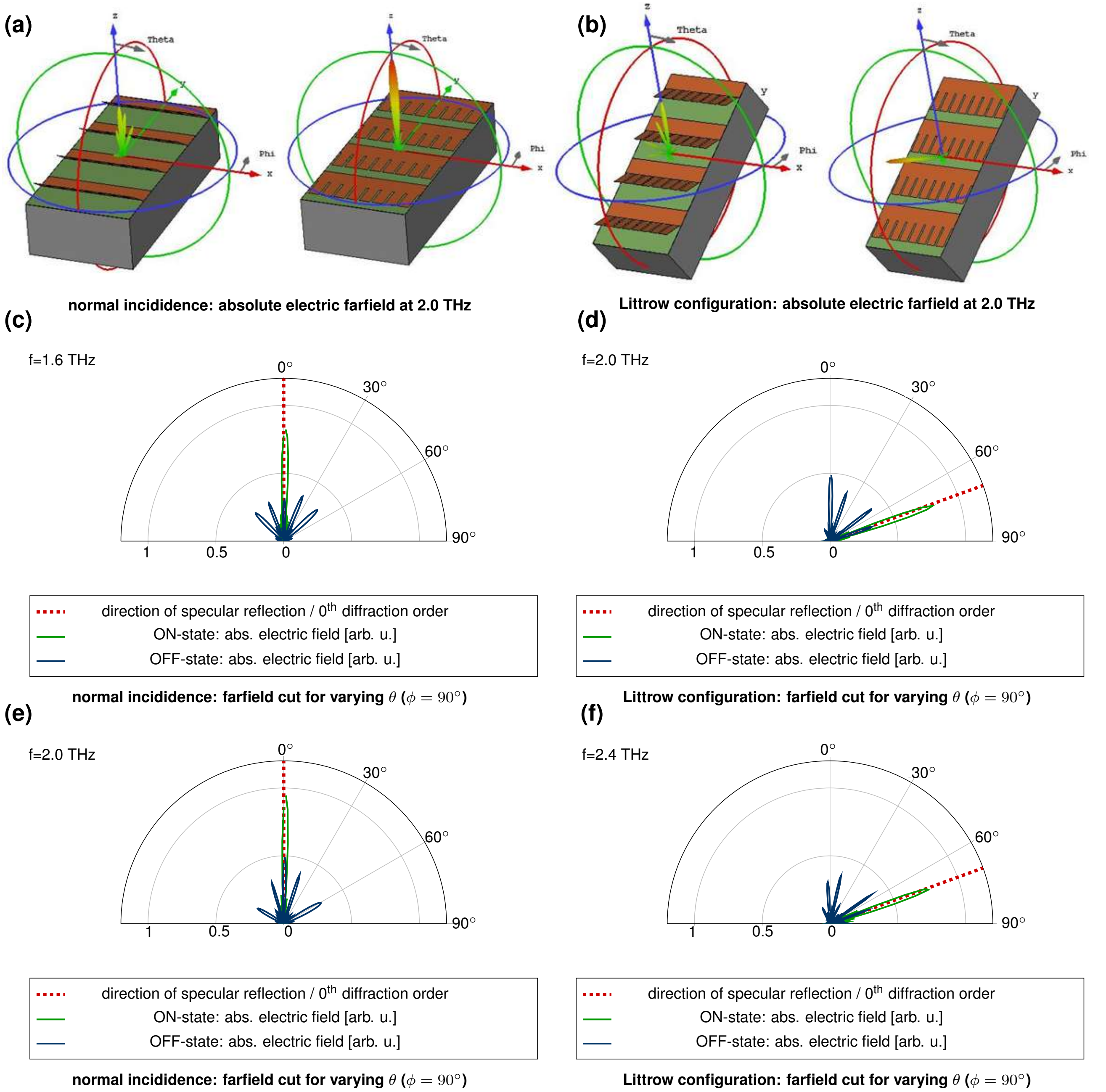

SI-Fig. 5. Simulated 3-D electric far-field after diffraction or reflection from a single pixel. A pixel is composed of 4 mirror rows and 8 mirror columns. (a) Electric far-field for normal incidence configuration at a frequency of 2.0 THz. (b) Electric far-field for Littrow configuration at a frequency of 2.0 THz. (c) Electric far-field for normal incidence configuration, evaluated in the cutting plane for $\phi = 90°$ and varying θ from -90° to +90°, which corresponds to the upper half-space in the y-z-plane in (a). The far-field was determined at a frequency of 1.6 THz. (d) Electric far-field for Littrow configuration, evaluated in the cutting plane for $\phi = 90°$ and varying θ from -90° to +90°, which corresponds to the upper half-space in the y-z-plane in (b). The far-field was determined at a frequency of 2.0 THz. (e) Same as in (c), but evaluated at a frequency of 2.0 THz. (f) Same as in (d), but evaluated at a frequency of 2.4 THz.

We observe in SI-Fig. 5(c) and 5(e) that the incident waves are reflected according to the reflection law, when all mirrors of the pixel are in the ON-state (flat on the substrate). This implies that all radiation is reflected back into the direction of the

incident wave, i.e. at an angle $\theta = 0°$ with respect to the $+z$-direction. Yet, when all mirrors of the pixel are in the OFF-state and inclined at 35°, the incident radiation is diffracted into multiple diffraction orders, as can be seen by the diffraction lobes in the far-field pattern. The diffraction lobes of different orders are symmetrically distributed around the z-axis. In the Littrow configuration in SI-Fig. 5(d) and 5(f), where the base line of the grating is rotated by 35° with respect to the z-axis, incident radiation is reflected according to the reflection law at $\theta = 70°$, when all mirrors of the pixel are in the ON-state. For all mirrors of the pixel in the OFF-state, the incident waves are diffracted from the grating into the different diffraction orders. The diffraction lobes are symmetrically aligned to an axis at $\theta = 35°$, the rotation angle of the base line of the grating.
From the far-field distributions, we calculated the modulation contrast as

$$C_{\mathrm{freq}} = \left.\frac{E_{\mathrm{ON}}^2 - E_{\mathrm{OFF}}^2}{E_{\mathrm{ON}}^2 + E_{\mathrm{OFF}}^2}\right|_{\mathrm{freq}} \tag{SI-2}$$

where E_{ON} and E_{OFF} denote the electric far field amplitude in the ON-state and OFF-state and the variable freq denotes the frequency, at which the electric field is evaluated.
For the normal incidence configuration, we obtained a modulation contrast $C_{1.6\,\mathrm{THz}} = 0.74$ and $C_{2.0\,\mathrm{THz}} = 0.76$. In the Littrow configuration, the modulation contrast of the THz-SLM was $C_{2.0\,\mathrm{THz}} = 0.73$ and $C_{2.4\,\mathrm{THz}} = 0.74$.
We concluded that pixels of 4×8 mirrors provide sufficient spatial modulation contrast to be used as THz-SLMs in coded aperture imaging. We note that the numerical calculations only consider approximations of the later fabricated THz-SLMs. For example, transverse flexure of the mirrors or inhomogeneous distribution of the inclination angle of the mirrors are not considered in the numerical evaluation. Nevertheless, the numerical investigation provides valuable insight into the physical properties of grating-based THz-SLMs and is indispensable for the design of such modulators. From the simulations, we derived guidelines for the design and fabrication of the THz-SLM presented in the main article.

2 Stress-Induced Bending of the Mirrors

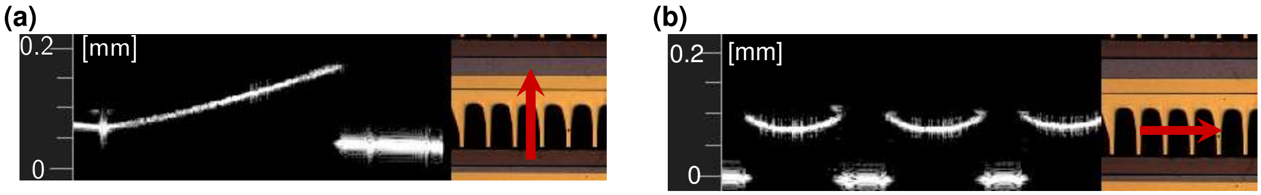

SI-Fig. 6. (a) Measurement of the longitudinal flexure of the mirrors by optical coherence tomography. The longitudinal flexure was measured in the direction along the red arrow in the inset. The mirrors were inclined at 35°. (b) Optical coherence tomography image of the transverse flexure of the mirrors, measured along the direction of the red arrow in the inset.

Analogue to the effects observed by Tyagi et al.[3], tensile stress in the upper Cr layer of a Cr-Cu-Cr multi-layer mirror and compressive stress in the middle Cu layer induces bending of the mirror. When the mirror is restrained to a support at one end, it inclines or declines dependent on the relative thickness of the three layer materials. In our case, the upper Cr layer is 7 nm thicker than the lower Cr layer in order to create a stress gradient that inclines the mirrors with respect to the substrate.
We measured the inclination angle and flexure of the cantilevers by optical coherence tomography (OCT). SI-Fig. 6 shows the measured images of the bent cantilevers and the ground electrodes. In SI-Fig. 6a), we measured the longitudinal flexure of the cantilevers along the direction that is indicated by the red arrow in the inset. The OCT image indicates an inclination angle of approximately 35°. At this angle, the tip of the cantilever is located 135 µm above the ground electrode. The cantilever mainly bends within a localized area around the support and extends in an almost linear shape outside the confined bending area.
Figure 6(b) illustrates the measured transverse flexure of the inclined cantilevers. The transverse flexure is measured along the direction indicated by the red arrow in the inset. The flat objects below the bent cantilevers constitute the unreleased parts of the Cr-Cu-Cr multi-layer structure that still sticks to the substrate. The transverse flexure prevents the cantilevers from curling up in the longitudinal direction and therefore maintains the linear longitudinal shape of the cantilevers outside the confined bending area around the support. The main article includes an additional scanning electron microscope (SEM) image in Fig. 2.

3 Hysteretic Actuation Concept

We applied the concept of hysteretic switching, as introduced by Canonica et al.[4], to independently actuate individual mirrors in the THz-SLM without addressing each mirror by a separate electrode.

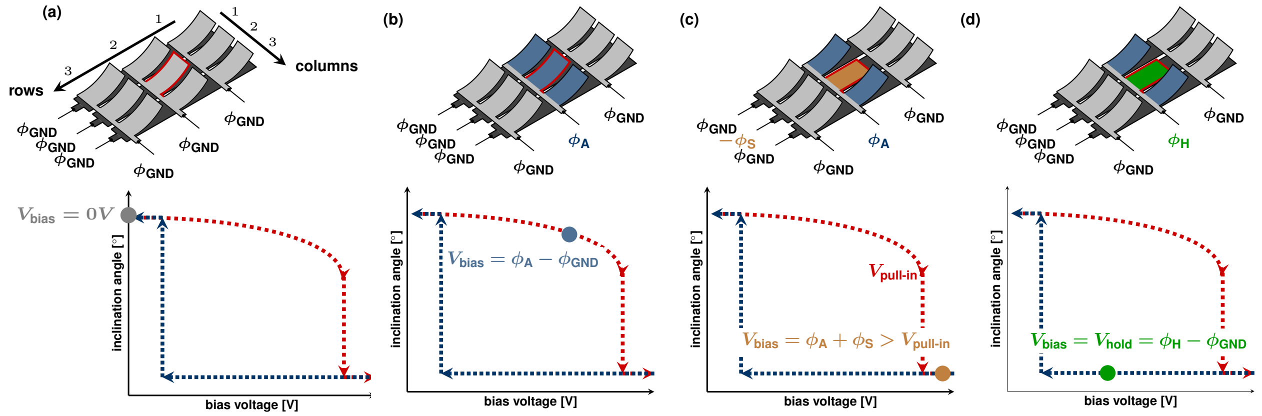

SI-Fig. 7. This schematic view illustrates the switching concept which utilizes the inherent hysteresis of the voltage dependent tip displacement of a single cantilever. The concept is taken from[4]. The rows are connected through the structure of the mirror electrode. The columns were realized through the ground electrode design.

The hysteretic switching concept can be explained by means of SI-Fig. 7 that displays the geometric arrangement of the bias and ground (GND) electrodes of the THz-SLM as well as the dependence of the deflection angle of an arbitrarily chosen individual mirror on the applied bias voltage.

As can be seen in SI-Fig. 7(a), the bias and ground electrodes lie in two vertically separated layers that are electrically insulated from each other. With respect to the bias electrodes in the top layer, all mirrors of a row are connected to the same bias electrode, while the bias electrodes of mirrors in different rows are not in electric contact. The opposite is the case for the ground electrodes in the bottom layer, where all mirrors of a column are connected by the same ground electrode, whereas the ground electrodes of mirrors in different columns are electrically insulated from each other. Since the applied bias voltage of an individual mirror is defined by the relative potential difference between bias electrode and ground electrode, we can tune the bias voltage by changing both the potential of the bias electrode and the ground electrode.

In order to understand why this electrode configuration enables the actuation of individual mirrors without affecting the position of its neighbors, it is necessary to take a look at the hysteretic dependence between inclination angle of the mirrors and the applied bias voltage. In SI-Fig. 7(a), all mirrors are unbiased and therefore inclined at the maximal inclination angle, as can be seen from the hysteresis. In the following, we arbitrarily choose the mirror in the second row and the second column to be actuated. For this purpose, we increase the potential of the bias electrode in the second row to ϕ_A, while keeping the potential of the ground electrode in the second column at $\phi_{GND} = 0\,V$, as illustrated in SI-Fig. 7(b). At this bias voltage of $V_{bias} = \phi_A - \phi_{GND} = \phi_A$, the addressed mirror is only slightly pulled towards the ground electrode, while the bias voltage of all other mirrors is zero. In a next step, the potential of the ground electrode in the second column is decreased to a value of $\phi_{GND} = -\phi_S$, at which the resulting bias voltage reaches the pull-in voltage $V_{pull\text{-}in} = \phi_A - (-\phi_S)$ of the mirror. Above this voltage, the addressed mirror snaps down to the ground electrode (see SI-Fig. 7(c)). We note that the bias voltage of all other mirrors, except for the mirrors in column 2, remains zero. Additionally, the bias voltage of all mirrors in column 2, except for the actuated mirror, stays at a bias voltage of $V_{bias} = \phi_{GND} - (-\phi_S) = \phi_S$, which lies well below the pull-down voltage. In a last step, we reduce the bias voltage to a hold voltage of $V_{bias} = \phi_H - \phi_{GND} = V_{hold}$ by simultaneously decreasing the potential of the bias electrode to ϕ_H and increasing the potential of the ground electrode to $\phi_{GND} = 0\,V$. As can be seen from the hysteresis in Fig. 7(d), the hold voltage is kept slightly higher than the voltage, for which the mirror snaps up and inclines to the maximal inclination angle. By repetition of the process, mirrors in the THz-SLM can be individually addressed without the need for connecting each mirror to individual electrodes that are electrically insulated from the electrodes of the other mirrors. In the experiment, we used an Arduino Mega 2560© micro controller and standard bipolar transistors (BD441) to provide the bias voltage.

4 Experiment

We investigated the THz-SLMs by use of terahertz time domain spectroscopy. We generated and detected the terahertz radiation with photoconductive antennas. We excited the photo carriers in the antenna substrate by 90 fs pulses at a center wavelength of 780 nm. The pulses were generated by a Ti:sapphire mode-locked laser (Tsunami Mode-Locked Ti:sapphire

Laser, Spectra-Physics). We adjusted the width of the terahertz beam using aluminum-coated off-axis parabolic mirrors with a focal length of 119.02 mm. By use of a lock-in amplifier (Model SR 830, Stanford Research Systems), we increased the signal-to-noise ratio of the measured terahertz signal.
For investigation of the spectral behavior of the THz-SLM, we calculated the spectrum of the terahertz pulses by Fast Fourier Transform of the time traces using the algorithm provided by Matlab©. For comparison of the spectra, we used zero-padding in the time domain to assimilate the number of sample points in the time traces, before calculating the Fourier transform. If necessary, we calculated interpolation points in the time domain to ensure that all Fourier spectra contained discrete entries at the same frequency points. The latter played an important role for the calculation of modulation contrasts.

References

1. Shrekenhamer, D., Watts, C. M. & Padilla, W. J. Terahertz single pixel imaging with an optically controlled dynamic spatial light modulator. *Opt. Express* **21**, 12507–12518 (2013).

2. Kappa, J., Schmitt, K. M. & Rahm, M. Electromagnetic behavior of spatial terahertz wave modulators based on reconfigurable micromirror gratings in littrow configuration. *Opt. Express* **25**, 20850–20859, DOI: 10.1364/OE.25.020850 (2017).

3. Tyagi, P. *et al.* Self-assembly based on chromium/copper bilayers. *J. Microelectromechanical Syst.* **18**, 784–791 (2009).

4. Canonica, M. D., Zamkotsian, F., Lanzoni, P., Noell, W. & de Rooij, N. The two-dimensional array of 2048 tilting micromirrors for astronomical spectroscopy. *J. Micromechanics Microengineering* **23**, 055009 (2013).
